# Hospital Meetings, &c.

**Published:** 1897-06-05

**Authors:** 


### HOSPITAL MEETINGS, &C.

### THE PRINCE OF WALES AT THE ROYAL LONDON OPHTHALMIC HOSPITAL.

On Friday, May 28th, the foundation-stone of the Royal London Ophthalmic Hospital's new buildings in City Road was laid with pomp and ceremony by H.R.H. the Prince of Wales on behalf of the Queen, who has been patron of the hospital for very many years. The way through the City, Old Street, and City Road was gay with flags and draperies, and hearty greetings everywhere met the Prince and Princess of Wales, with whom was Princess Victoria. Punctually at the very stroke of four o'clock the Royal party arrived at the site of the new buildings, already in a fairly forward state, the walls having reached the second storey. Their Royal Highnesses were here received by Sir John Lubbock, M.P. (president of the hospital), Mr. H. P. Sturgis (the chairman), Mr. J. Deacon (treasurer), and others, and conducted to a reception-room, where members of the staff and others were presented, thence proceeding to the marquee erected in what will presently be a large out-patient department. Here under a canopy of scarlet cloth hung suspended the "foundation stone." Those who were responsible for the arrangements are truly to be congratulated on the success of their efforts, for the scene in the tent was a most effective one. Bouquets were presented to the Princess of Wales by Miss Rosamond Smith, and to Princess Victoria by little Miss Tweedy, a pretty picture in quaint white frock and Victorian bonnet.

In a short address Sir John Lubbock summarised the events which had led to the imperative need for a new building, and gave the history of the old hospital, now in Blomfield Street, Moorfields. It was the first institution "for the relief of the poor afflicted with eye disease" established in Europe, and was instituted by certain generous "Gentlemen of the City of London" in 1804. The present buildings, Sir John explained, were totally inadequate for the heavy work carried on within its walls, and a suitable site for a new hospital had therefore been obtained, to properly carry out the plans for which a sum of £85,000 would be necessary.

The Prince of Wales replied: Ladies and Gentlemen,—As Sir John Lubbock has stated, this institution was founded in 1804 by a number of gentlemen in consequence of the great sympathy aroused by the terrible sufferings of the men of the Army and Navy belonging to the force employed in the Egyptian campaign under Sir R. Abercromby, when many of our soldiers and sailors were invalided home. It is a significant fact that while in this campaign in the early part of the present century ophthalmia committed great ravages—out of a battalion of 700 men there were 636 cases, of which 40 became totally blind in one eye, and 50 totally blind in both eyes—in the campaign of 1882, although there were 1,800 cases of ophthalmia after the Battle of Tel-el-Kebir, not one of those affected lost his sight, which was entirely due to the exercise of proper precautions. The Queen began her long connection with this hospital in 1836, before she succeeded to the Throne, by becoming its patron. The importance of this hospital is not so much in the number of beds, which are only 100, as in the extraordinary number of out-patients, amounting occasionally to as many as 550 in one morning. Altogether it is estimated that over 1,000,000 persons of the poorest class, all suffering from eye disease, have been treated in this hospital since the Queen ascended the Throne. Patients have come from all parts of the United Kingdom, and even from the colonies. Regarded as an important school of ophthalmic surgery, there is probably not one surgeon of eminence in the United Kingdom and the colonies who has not studied at this hospital, while qualified medical practitioners come here from all parts of the world—from Canada, a large number from the United States, and some even from Peru—to study ophthalmic science and surgery. The present buildings in Moorfields are over 70 years old, and are very defective in the necessary air, space, and light required for the treatment of patients. The new hospital, which it is hoped will soon be completed, will greatly benefit those who are suffering from eye disease. The cost will, however, be very heavy and beyond the resources of the Hospital Committee, unless they succeed in obtaining more liberal support than has hitherto been accorded them. It, therefore, gives the Princess, my daughter, and myself great pleasure to come here to-day, and I feel that in doing so I am performing an act to which I have been deputed by her Majesty the Queen. I know that it is her great and earnest wish that this hospital may be prosperous and successful in every way; and I venture to think that we may also regard this event as a fitting memorial of this particular year, when the Queen celebrates the sixtieth year of her reign.

The foundation-stone was then lowered into its place, tapped thrice with the mallet by the Prince, and declared in ancient formula to be "well and truly laid." Dedicatory prayers were said by the Bishop of London, and finally a number of purses in aid of the Building Fund were presented to the Princess, the ceremony concluding with a few words of grateful thanks to the Royal visitors for their presence from Mr. Sturgis and the Lord Mayor on behalf of the hospital.

### THE PRINCE OF WALES AT GUY'S HOSPITAL.

On Wednesday afternoon, May 26th, H.R.H. the Prince of Wales visited Guy's Hospital, of which his Royal Highness is President, to open the new Medical School Buildings. Arriving punctually at four o'clock, the Prince was received by Mr. Cosmo Bonsor, treasurer, and Dr. Perry, superintendent of the hospital, and accompanied by H.R.H. the Duke of Cambridge proceeded to the court-room, where he presided over a meeting of the general court, and a number of governors and prominent supporters of the hospital were presented to him.

A statement made by Mr. Bonsor showed that in answer to the Prince's appeal for a re-endowment fund for the hospital a total amount or £194,000 had been received. While looking forward to the future with confidence, the treasurer estimated that an additional £15,000 per annum would be still required to secure the full advantage of the hospital to the sick and suffering poor of South London. It had been decided to re-open one of the closed wards, and the most urgent present need was, in the unanimous opinion of the House Committee, the erection of a nursing home, for no further wards could be opened until increased accommodation was provided for the nursing staff.

The business of the court over, the Prince was escorted by the governors to a platform erected at the south end of the colonnade, where the members of the medical and surgical staff were presented to his Royal Highness, and an address of welcome read by Mr. Howse, the senior surgeon. Then proceeding through the hospital garden to the new buildings, after inspecting the laboratories and class rooms, the Royal party entered the splendid theatre, while a choir of nurses and students sang "God Bless the Prince of Wales" with admirable effect. Here Dr. Pye Smith, the senior physician, invited the Prince to declare the buildings open, explaining in a brief speech their objects, and the great indebtedness of the school to the architect, Mr. Woodd, and Dr. Shaw, Dean of the medical school. He pointed out the difficulty of obtaining funds for the purposes of the school, which had of course no claim on money subscribed for the hospital, from which it was a distinct institution. In the absence of Government grants or private gifts the staff had but one thing to do if they wished to maintain their school at a proper level, and this was to find the money themselves. "May I invite you, sir," concluded Dr. Pye Smith, in Harvey's words, "to enter these unattractive rooms not without fitting reverence, for here the secrets of life and death are sought out, and here also the benefit of mankind is promoted."

The Prince replied: "Your Royal Highness, my Lords, Ladies, and Gentlemen,—I have much pleasure in declaring this building open.

"It is a matter of congratulation with me that having taken some share in the attempt to re-endow this great hospital—an attempt which indeed seems to give promise of being crowned before many years with success—I am now enabled to give a further proof of my interest by inaugurating this considerable extension of its medical school.

"I understand that the building which I have just declared open is to be used in great part for the study of those sciences which have for their object the observation of the natural laws of life. It was in such observations that your distinguished physician, Sir William Gull, first won renown. Nor is it possible to over-estimate the value of such work in the investigation and treatment of disease. One thing I would venture to impress upon our students, namely, that in endeavouring to follow in the footsteps of the great and good men whom Guy's delights to honour, they should cultivate that gentle and humane spirit which, not confined to any one school, is the best possession of the medical faculty.

"I have made careful inquiries, and have every reason to believe that whenever experiments upon animals are performed in this school, they are undertaken with the object of promoting advances in medicine and surgery which are likely to be of benefit to suffering humanity, and I have satisfied myself that such experiments are conducted under strict supervision, by highly qualified investigators, and that in practice the only operations performed upon animals which are not in a condition of complete anæsthesia are inoculations and hypodermic injections.

"Students enter a medical school with their characters still unformed, and they are easily influenced by their surroundings. I am glad to believe that in their early studies our students will not be left without due guidance and humane example.

"Looking back upon the history of this school, one cannot but admire the wonderful powers of observation which enabled such men as Astley Cooper, Bright, Addison, Hodgkin, and Gull, with but slender aid from scientific apparatus, to add so largely to the sum of human knowledge. I need hardly remind you that more than one of these great workers in your profession has had his name perpetuated in connection with the discoveries he made in the wards of Guy's Hospital—discoveries which paved the way for the more enlightened treatment of some of the most frequent, and yet most fatal, diseases to which man is subject. That harvest has been gathered, and for the present and future generations it remains, with more exact appliances and more delicate apparatus provided by the sister sciences, to seek on other fields to emulate their illustrious predecessors' example. To this end are needed ampler buildings, specially-designed rooms, and complicated mechanical contrivances, all of them involving additional expenditure. Your senior physician has made it clear to all how, relying upon themselves, the staff of the medical school have erected this building. I may be permitted to emphasise the fact, to which Dr. Pye-Smith has alluded, that the present building is but an instalment of a more extensive design which is to be completed as soon as funds are forthcoming.

"I would venture to express a hope that this day may not be long delayed, and that when the building is completed, room will be provided to adequately display the unique collection of wax models which so much interested me when first I visited your museum.

"The medical staff have expended as much as they safely can, and it is to men of wealth and philanthropic aspirations that we confidently look for further assistance. Let such men once realise that money given for the purposes of medical education directly benefits humanity, and I cannot doubt that that spirit, which has prompted the British people to provide by voluntary effort what in other countries is provided by the State, will prove effective in the present need.

"On you students of medicine—and a medical man, as Dr. Wilks has said, should be a student till he dies—it devolves so to order your life's work that you make the best use of the improved opportunities thus provided, and to take care that the great profession to which you have aspired to belong shall, when you leave it, stand as high in the service and in the affection of the public as it does at the present time."

The Prince's speech was received with much applause, and prayers having been said by the Bishop of Rochester, Mr.

Cosmo Bonsor moved a vote of thanks to his Royal Highness, endorsed with unmistakable enthusiasm by the audience. The newly-reopened ward was named the "Queen Victoria Ward" by the Prince, who declared himself in hearty accord with this step, and then followed by the strains of the National Anthem sung by the choir and the visitors, his Royal Highness returned along the colonnade, lined with sisters, nurses, and patients, to the reception platform, where he took leave of the governors and staff.

Tea was afterwards served to the visitors, who numbered some 3,000, in marquees in the grounds, and later Mrs. Cosmo Bonsor distributed prizes and medals to successful students in the medical school.

### LONDON HOMŒOPATHIC HOSPITAL.

In order to commemorate the Diamond Jubilee by completing the building fund for the new hospital a festival dinner was held at the Hotel Cecil on Wednesday, 26th ult., Lord Emlyn, treasurer of the hospital, presiding. The Chairman, in proposing the toast of the evening, congratulated homœopaths on the immense progress which homœopathy had made during the Queen's reign. In 1837 there was but one homœopathic physician in the United Kingdom, Dr. Quin, the founder of the London Homœopathic Hospital, while at the present time there were homœopathic practitioners in every important town: the hospital in 1849 had 25 beds, now it had 100; and it had treated 156 in and 906 out patients, in its first year, as against 1,031 in and 14,500 out patients during 1896. Even during the last ten years the increase had been most remarkable, as the average for the ten years preceding 1896 was 674 in and 8,000 out patients. It had been said that there were but few homœopaths in England. In order to disprove this all his hearers had to do was to show any doubters over the hospital, and to tell them that it was a freehold, that it had no debt, and that it had cost over £50,000, that £50,000 having been raised by homœopaths in five years. The dinner resulted in a collection amounting to the large sum of over £7,000.

### THE HOSPITAL FOR SICK CHILDREN.

The Duke of Fife presided at the forty-fifth annual meeting of The Hospital for Sick Children, which was held on Wednesday, 26th ult., at the hospital, Great Ormond Street, W.C. The meeting was well attended, amongst those present being Sir Andrew Clarke, Lord Gort, Mr. Arthur Lucas (chairman), Colonel Wilkinson, Messrs. John Murray, C. E. H. Chadwyck Healey, Q.C., G. P. Eyre, Charles Lindo, Hugh L. Arbuthnot, Cecil H. Russell, and A. C. Norman.

The Chairman, in opening the proceedings, said that although the financial condition of the hospital was fairly satisfactory, yet still there was again this year a decrease in the amount of annual subscriptions and of donations received. The decrease in the donations he did not feel so concerned about, as they could be accounted for by the fact that there was no dinner last year, but he thought that every effort should be made to ensure there being no falling off in the annual subscriptions.

The report for the year 1896 was adopted on the motion of Mr. Arthur Lucas, seconded by Colonel Wilkinson. The mover, in the course of a brief speech, stated that the expenditure, including that on the Convalescent Home at Highgate averaged yearly no less than £14,400—towards which their regular income came to about £10,200, leaving about £4,200 to be made up from irregular sources each year. Last year a most careful inquiry had been made into the management of the hospital, and the result had been that they were able to save about £300 to £400 a year. So careful was this inquiry that the management had now organised a system by which every piece of lint or every gallon of carbolic acid could be traced, so that if there was any extravagance the person responsible for it could be brought to book. Speaking of the convalescent home, he said that that home was, after all, situated in London, and he was afraid that soon they would have to take steps to find another.

On the motion of Mr. Arthur Lucas, seconded by Mr. John Murray, it was resolved that a congratulatory address be presented to the Queen on behalf of the court of governors. The address pointed out that Her Majesty's long-continued patronage of the Hospital for Sick Children—the pioneer of children's hospitals throughout the world—was only one among the innumerable proofs of her unvarying sympathy with suffering, distress, and helplessness, which had become a household word among all her subjects.

The re-election of the honorary officers and the passing of votes of thanks completed the business of the meeting.

### MIDDLESEX HOSPITAL.

A quarterly general court of the Middlesex Hospital was held on Thursday, 27th ult., the Duke of Cambridge in the chair. During the course of the ordinary business the court was made special for the purpose of passing a congratulatory address to the Queen.

Sir Ralph Thompson (chairman of the Weekly Board), in moving the adoption of this address, said that the gratitude felt by the hospital for the blessings of her Majesty's reign took a personal form, because not only was the Queen a patron of, and a subscriber to, the hospital, but the members of the Royal Family had always been particularly kind to it.

Dr. William Cayley, senior physician, seconded.

The Duke of Cambridge, after congratulating the hospital on having Sir Ralph Thompson as their chairman, said that in nothing was the Queen's reign more conspicuous than in the advance which had been made by medical science. He was glad to see that the older hospitals were coming to the fore on the present occasion, because the newer hospitals were always very anxious to bring themselves into public notoriety; while the older ones, not feeling the want of such notoriety, kept in the background, with the result that they were sometimes overlooked.

The address concluded as follows: "We feel assured that no one of the many blessings which have fallen upon the nation through your Majesty's wise administration of its affairs has tended to shed a greater lustre upon the Throne, or has more effectually secured the affection of your subjects, than the benefit derived by the sick poor from the tender solicitude and womanly sympathy ever displayed by your Majesty in their behalf. Your Majesty's reign will be conspicuous, not only for the great advance in the science of medicine and for the establishment of a body of highly-trained nurses, but for the endeavours made, under the stimulus and encouragement of your Majesty's bright example, by members of the Royal Family, to alleviate the sufferings of the sick, which has now culminated in the noble and generous effort, by his Royal Highness the Prince of Wales, to raise such a lasting fund for the benefit of the hospitals of London as will render the sixtieth year of your Majesty's reign specially marked in their annals. We fervently pray that your Majesty may long continue to rule over the destinies of this realm, and to live in the hearts of your faithful and loving subjects."

The motion was put from the chair, and the address was unanimously adopted. The address having been signed by the Chairman, on behalf of the president, the vice-presidents, governors, &c., of the hospital, the Duke of Cambridge consented, at the request of Sir Ralph Thompson, to present it to her Majesty.

The proceedings shortly afterwards terminated.

HOSPITAL FOR CONSUMPTION, BROMPTON.

The annual court of governors was held in the board-room of the hospital on 27th ult., the Earl of DERBY in the chair. The Chairman, in moving the adoption of the report, stated that the committee had decided to establish a country branch and convalescent home in connection with the hospital as a memorial of her Majesty's Diamond Jubilee, and earnestly commended this work to the consideration of the friends of the charity. The amount required for this purpose was, as nearly they could estimate, £20,000.—Sir Patrick Talbot seconded, and the report was adopted.—From the report we find that 1,595 in-patients and 13,416 new out-patients had been treated in 1896. The annual subscriptions showed a slight increase, but the donations and legacies had decreased. A committee of management was appointed; Mr. George Herring was elected an honorary governor, and the proceedings closed with a vote of thanks to the chairman.

